# Exploring the Consistent Roles of Motor Areas Across Voluntary Movement and Locomotion

**DOI:** 10.1177/10738584241263758

**Published:** 2024-07-23

**Authors:** Nicolas Fortier-Lebel, Toshi Nakajima

**Affiliations:** 1 Département de neurosciences, Département de médecine, Centre interdisciplinaire de recherche sur le cerveau et l’apprentissage, Groupe de recherche sur la signalisation neurale et la circuiterie, Université de Montréal, Montréal, Canada; 2 Department of Physiology, Faculty of Medicine, Kindai University, Osaka-Sayama, Japan

**Keywords:** motor cortex, premotor area, monkey, cat, voluntary movement, gait modification, locomotion, motor sequence

## Abstract

Multiple cortical motor areas are critically involved in the voluntary control of discrete movement (e.g., reaching) and gait. Here, we outline experimental findings in nonhuman primates with clinical reports and research in humans that explain characteristic movement control mechanisms in the primary, supplementary, and presupplementary motor areas, as well as in the dorsal premotor area. We then focus on single-neuron activity recorded while monkeys performed motor sequences consisting of multiple discrete movements, and we consider how area-specific control mechanisms may contribute to the performance of complex movements. Following this, we explore the motor areas in cats that we have considered as analogs of those in primates based on similarities in their cortical surface topology, anatomic connections, microstimulation effects, and activity patterns. Emphasizing that discrete movement and gait modification entail similar control mechanisms, we argue that single-neuron activity in each area of the cat during gait modification is compatible with the function ascribed to the activity in the corresponding area in primates, recorded during the performance of discrete movements. The findings that demonstrate the premotor areas’ contribution to locomotion, currently unique to the cat model, should offer highly valuable insights into the control mechanisms of locomotion in primates, including humans.

## Introduction

Primates, including humans, have well-differentiated primary motor and premotor cortical areas. Since Evarts pioneered single-neuron recordings from awake head-restrained monkeys in the 1960s, accumulating evidence has demonstrated that each motor area plays characteristic roles in preparing and executing discrete movements, such as reaching for an object, manipulating a controller, or pressing keys based on visual and somatic information.

Locomotion—often classified as a type of highly automatic behavior distinct from discrete movements—is subject to voluntary control, which becomes increasingly critical with more challenging environments. For example, precise voluntary gait modifications are undoubtedly necessary to walk up a mountain trail or around a cluttered room, where low obstacles and preferential stepping targets abound. Experimental evidence that motor areas are essential for regulating such gait modifications was first provided by Liddell and Phillips (1944): following bilateral transection of the pyramidal tracts, cats completely lost the ability to step from rung to rung on a horizontal ladder, although their walking performance on a level floor was largely preserved. However, the main interest in locomotion research for the following decades stayed on the circuitry that generates stereotypical gait patterns, which consists of the central pattern generators in the spinal cord and the brainstem locomotor centers. Armstrong and Drew (1984) were among the pioneering groups that studied how the primary motor area in the cat controls locomotion at the single-neuron level. Single-neuron evidence for the roles of the premotor areas in gait modification have also recently emerged in cats. Notably, these recordings have compensated for a general lack of information about the precise roles of the primate and human motor areas in controlling locomotion.

In this article, we aim to synthesize evidence on the parallel contributions of cortical motor areas to voluntary movements in primates and gait modifications in cats. To this aim, we review evidence supporting the notion that separate motor areas are responsible for specific aspects of motor control: muscle-oriented control (primary motor cortex [M1]), coordination of multiple effectors (supplementary motor area [SMA]), and inhibition-based control (pre-SMA). Specifically, major anatomic connections and lesion and stimulation studies in the primate with clinical findings in humans are briefly outlined. We then consider the function of each area in more detail with reference to single-neuron recordings on primates. We subsequently explore the cortical areas in cats with close anatomic and physiologic similarities to these three areas in primates. We propose that activity in the cat motor areas during gait modification are compatible with the basic functions of their primate analogues. We conclude that multiple motor areas are each uniquely involved in controlling discrete movement and voluntary gait modification. Finally, we devote some space to the dorsal premotor area (PMd) in the primate.

## Immediate Execution of Voluntary Motion: M1 and Area 4γ of the Cat

**Figure 1.**
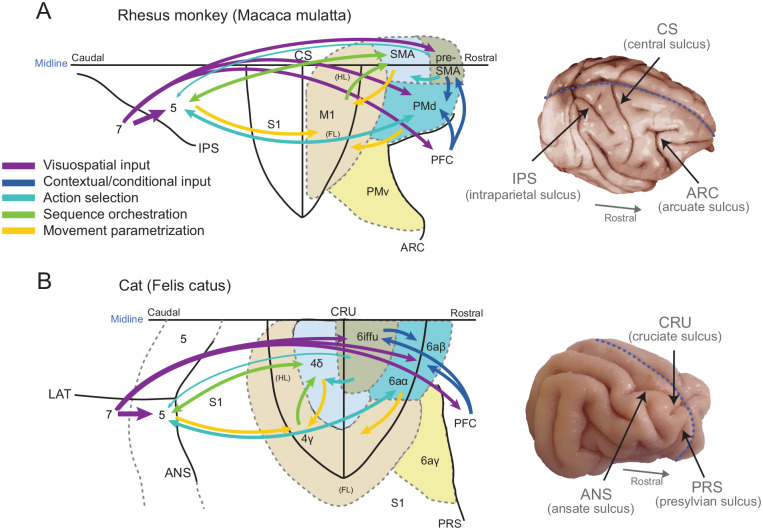
Frontal motor areas of the primate and cat. A proposed flow of the common key stages of visuomotor transformation unfolding during goal-oriented voluntary motor sequences (from cutting vegetables to negotiating a curb). Information flow (colored arrows) is based on studies of neuronal activity, microstimulation, and corticocortical connectivity (see main text) in the cat (A) and primate (B), with an emphasis on the evolution of visual information into motor commands. On the left, flattened views of the cortical surfaces with the central (A) and cruciate (B) sulci unfolded. Shading indicates anatomofunctional similarities between motor areas of the two species. On the right, dorsolateral views of the brains, with dotted blue line indicating the midline. Modified from Drew and others (2023). ANS = ansate sulcus; ARC = arcuate sulcus; Cing = cingulate cortex; CRU = cruciate sulcus; CS = central sulcus; FL = forelimb representation; HL = hindlimb representation; IPS = intraparietal sulcus; LAT = lateral sulcus; PFC = prefrontal cortex; PMv = ventral premotor cortex (not discussed in this review); PRS = presylvian sulcus.

In humans, nonhuman primates, and other mammals, M1 is the core area responsible for generating voluntary movements (Lemon 2008; Omrani and others 2017). It is bordered at the fundus of the central sulcus by the primary somatosensory cortex (S1) and meets anteriorly with SMA and the caudal aspect of the dorsal and ventral premotor areas (Figure 1A). Through bidirectional connections with these areas, as well as more distant afferents from area 5 of the posterior parietal cortex, M1 integrates higher-order motor commands and modulating inputs. It then projects directly and massively to the spinal cord through the corticospinal tract (CST), where it exerts a strong, highly fractionated influence on the contralateral skeletal musculature through connections with the spinal motoneurons that are both direct (monosynaptic, strictly in primates) and indirect (Lemon 2008).

Electrical stimulation in M1, which evokes movement at a very low threshold, reveals a complete and intricately detailed contralateral motor somatotopy organized in humans and macaques from the foot to the head along a mediolateral axis. Microstimulation of individual sites in the arm or leg representation elicits focal movements restricted to biomechanically coupled joints (Schieber 2001). In humans, M1 is critical for voluntary limb control, as exemplified by the common finding of somatotopically coherent paresis following stroke involving part of its territory. Similar deficits are observed in lesion studies (Passingham and others 1983) and reversible inactivation (Goldring and others 2022) of M1 in primates.

**Figure 2.**
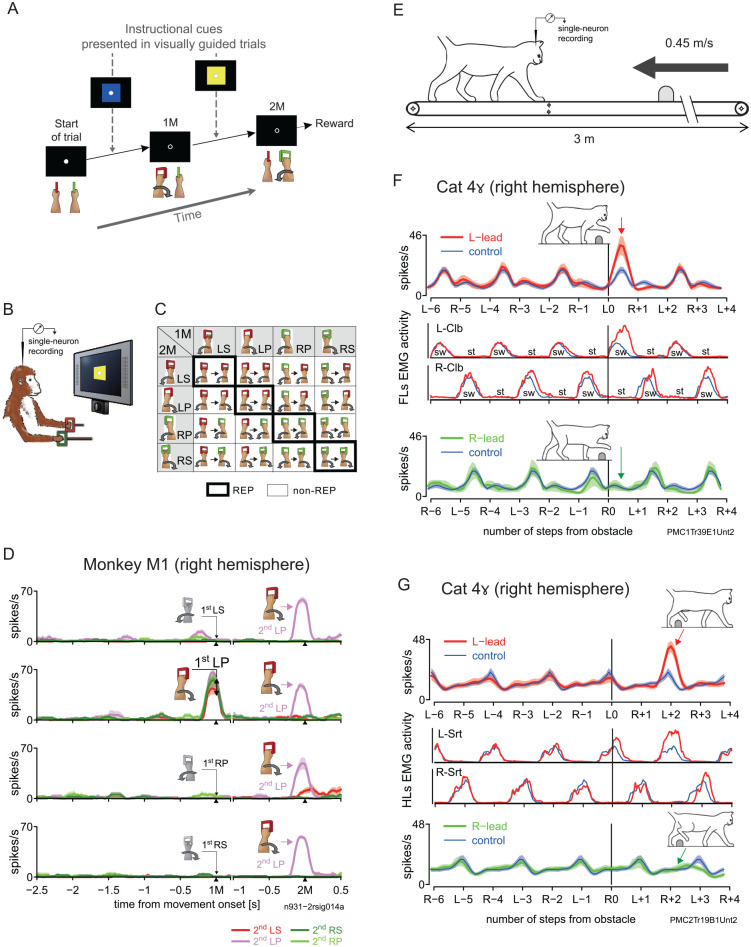
Behavioral tasks and single-neuron activity in the primary motor cortex. (A) Sequence of events in a sample trial block of the sequential motor task, where monkeys were required to memorize and perform a particular motor sequence (left forearm pronation–right forearm pronation, bottom row; Nakajima, Hosaka, and Mushiake 2022; Nakajima and others 2013). The first (1M) and second (2M) movements in the sequence were initially instructed with colored cues (top row) and memorized thereafter. (B) Experimental setup. The monkey turned manipulanda while fixating on the center of a screen throughout a trial. Eye position was monitored with an infrared camera. (C) Classification of motor sequences performed by the monkeys. A matrix of 16 motor sequences determined the order of pronation or supination of the forearm(s). Cells in the matrix indicate repetition sequences (REPs; thick borders) and nonrepetition sequences (non-REPs; thin borders). LP = left forearm pronation; LS = left forearm supination; RP = right forearm pronation; RS = right forearm supination. (D) Activity of a typical M1 neuron during the analysis interval, which starts 2.5 seconds before the 1M and ends 0.5 seconds after the 2M in memory-guided trials. For display purposes, we adjusted for variation of the interval between the movements by inserting a gap between 200 milliseconds after the 1M onset and 1 second before the 2M. The perievent histograms for instantaneous firing rates (mean ± SE) for each sequence were sorted by the 1M and then color coded with respect to the 2M for the same analysis interval. Occurrence of the 1M related to activity enhancement (LP for this neuron) is indicated with colored cartoons, whereas the other 1Ms are illustrated in grayscale. The double-headed arrow indicates activity range at the occurrence of 1M. On the right side, the 2M related to activity enhancement (LP) is likewise illustrated with colored cartoons. (E) Experimental setup for a gait modification task devised for cats (Nakajima, Fortier-Lebel, and Drew 2022; Nakajima and others 2019). The cat stepped over an obstacle attached to a second belt while walking on a treadmill at a speed of 0.45 m/s; the obstacle moved at the same speed as the treadmill. (F, G) Sample neurons recorded from the right primary motor cortex (area 4γ) in the cat. For cats, all the recordings were made from the right hemisphere. Instantaneous firing rate of each neuron (mean ± SE) is illustrated for the situations when the forelimb contralateral to the recording site was the first to step over the obstacle (red trace in top row, left lead condition) and when the ipsilateral forelimb was the first (green trace in bottom row, right lead condition). Data are plotted along the number of forelimb steps with respect to the onset of the first step that passed over the obstacle (L0 in left lead or R0 in right lead condition) and superimposed on activity recorded during control locomotion without the obstacle (blue trace). The analysis period consisted of 3 step cycles (6 steps) before the obstacle and 2 step cycles (4 steps) after it. Below the abscissa, the side of the forelimb (L or R) that initiated each step is indicated with the associated number of the step (negative values for steps before the obstacle, positive values for steps after). Middle rows show forelimb (F) or hindlimb (G) electromyography activity in the left-lead condition. Cartoons depict the cat at the times indicated with associated arrows. Panel F is modified from Figure 14F of Nakajima and others (2019). Clb = cleidobrachialis (forelimb flexor); Srt = sartorius (hindlimb flexor); st = stance phase; sw = swing phase.

Single-neuron recordings in primates during discrete voluntary arm movements have shown that the activity of M1 neurons within the representation of the performing limb closely correlates in time and magnitude with low-level parameters of the movements, including muscle activity (Evarts 1968) and movement direction (Georgopoulos and others 1982). Such a relationship can be observed in Figure 2. In this experiment, Nakajima and others (2013; Nakajima, Hosaka, and Mushiake 2022) recorded in SMA, pre-SMA, and PMd, as well as M1 (unpublished data), of macaque monkeys performing multiple motor sequences, each consisting of two movements separated by a delay. Each movement involved pronation or supination of either arm (Figure 2A–C). In M1, single neurons typically responded to a specific movement of the contralateral limb. For instance, the representative neuron recorded in right M1 only and always discharged immediately before and during left arm pronation (Figure 2D).

Combining the specificity of individual neurons, M1 contains a neuronal population encoding of limb kinematics (Georgopoulos and others 1986), an insight that has led to the proof-of-concept development of robotic arm neuroprostheses in humans (Collinger and others 2013). However, while the encoding of explicit physical quantities in egocentric reference frames is a robust feature of M1 activity, these vary across tasks and studies, pointing to the complexity of the computational mechanisms through which M1 integrates higher-order inputs to command movements (Omrani and others 2017; Scott 2008). Mounting evidence in the last decade is favoring a dynamical system perspective, in which the evolving neural state of M1 (usually interpreted as a high-dimensional trajectory of the population activity across time, then amenable to various analytic approaches) converges through trajectory-altering external inputs and trajectory-constraining internal dynamics toward a state signaling a given movement (Churchland and others 2010; Shenoy and others 2013). This perspective is likely to offer mechanistic insights relevant to motor control beyond discrete movements—for instance, how preparatory activity in other motor areas can silently shape future M1 commands without unduly altering the immediate ongoing motor output (Kaufman and others 2014).

Still, as we will contrast with the motor areas of the next sections, a core functional characteristic of M1 is the close relationship between its activity (be it peak discharge rate or potent neural trajectory) and movement. With its functional and synaptic connectivity with the downstream motor apparatus, all point to its role as a direct controller of voluntary movements.

As hinted in the introduction, this role of M1 extends to the control of walking. In humans, clinical evidence has shown that stroke of the leg representation leads to paretic gait with difficulties adapting the stepping to environmental challenges (Said and others 2005). More severely, CST transection causes a sustained inability to walk (Nathan 1994), although one should note that premotor areas are major contributors to the human CST (Usuda and others 2022). While targeted lesions of M1 or the CST in quadrupedal primates (Courtine and others 2005) and cats (Liddell and Phillips 1944) disrupt but do not preclude basic locomotion, evidence in cats shows a severe loss of the ability to proactively adapt their gait. This includes overcoming low obstacles (Drew and others 2002) or stepping precisely in other complex environments (Beloozerova and Sirota 1993; Liddell and Phillips 1944).

Regarding the neural mechanisms through which M1 performs this function, the most comprehensive experimental evidence comes once again from studies in the cat. Indeed, single-neuron recordings in area 4γ (Figure 1B; the cat’s M1) have shown that 4γ neurons are rhythmically active during locomotion, each discharging in synchrony with a specific phase of the contralateral forelimb’s or hindlimb’s step cycle (Armstrong and Drew 1984). Frequent in CST-projecting neurons, this rhythmic activity is modified during voluntary gait modifications (Beloozerova and Sirota 1993; Drew 1993; Drew and others 2002) and matches the changes of activity of functionally synergistic muscles in the contralateral limb as they perform their part of the gait modification (Krouchev and Drew 2013). Examples of 4γ single-neuron activity during the crossing of an obstacle are displayed in Figure 2F and 2G, where one neuron in the contralateral forelimb representation increases its flexor-related discharge during the swing phase of that limb over the obstacle, while another in the hindlimb representation strongly discharges during its crossing.

In parallel, stimulation of area 4γ or the pyramidal tract during locomotion produces smooth phase-dependent changes in the muscle activity of the represented limb (Rho and others 1999), suggesting a system that supports the timely integration of discrete voluntary commands from 4γ into the gait. Since longer trains of stimulation can even reset the gait cycle (Rho and others 1999), 4γ appears capable of altering the temporal structure of the gait, as would be needed for major adjustments to environmental challenges.

Because area 4γ is critical to accurate reaching (Martin and Ghez 1993) and voluntary gait modifications (see previous references) and given that single-neuron activity maintains its quantitative relationship with the activity pattern of specific muscles in the two behaviors (Yakovenko and Drew 2015), the properties of M1 control over reaching movements and gait modifications appear fundamentally similar (see Georgopoulos and Grillner 1989).

While most studies on the cortical control of locomotion in humans and primates have focused on basic walking, there is good evidence that M1 contributes to voluntary gait control similarly to the cat 4γ. In primates, contralateral hindlimb kinematics during simple walking (Fitzsimmons and others 2009) and, recently, the stepping over an obstacle (Xing and others 2022) can be decoded from M1 population activity. In humans, coherence studies between muscle and electroencephalographic activity have revealed a corticospinal influence over muscle activity during visually guided gait modifications (Spedden and others 2019). This agrees with transcranial magnetic stimulation of M1 unveiling a phase-dependent influence over leg muscle activity during walking, particularly when precise steps are necessary (Schubert and others 1999). Thus, integration of descending M1 commands into the human gait appears to follow similar fundamental mechanisms as in the cat.

## Cortical Regions Responsible for Interlimb Coordination: Primate SMA and Feline 4δ

The primate SMA is located in the medial frontal lobe, anterior to the hindlimb representation of M1 (Figure 1A). Electrical stimulation shows somatotopy within SMA: by probing sites along its caudal-to-rostral border, movements sequentially involve the contralateral hindlimb, trunk, contralateral forelimb, and face (Mitz and Wise 1987).

SMA, however, differs from M1 in the efficacy of stimulation and the nature of evoked responses. First, it generally requires a stronger current than M1 to induce movement (Luppino and others 1991; Mitz and Wise 1987). This is accounted for by SMA’s lower ability to directly drive spinal motoneurons; it mainly projects to the intermediate zone of the spinal cord, sending fewer projections to the ventral horn than does M1 (Maier and others 2002). Second, microstimulation within SMA often induces multijoint movements (Mitz and Wise 1987), which can involve bilateral limbs or forelimb and hindlimb (Luppino and others 1991). When compared with M1, this coarse somatotopic organization mirrors the capability of SMA to coordinate multiple limbs and joints, as described later.

Although damage to SMA does not lead to sustained paralysis when M1 is spared, it causes various symptoms: global reduction in spontaneous movements of the contralateral limbs (Laplane and others 1977) and decreased muscle tone (Travis 1955), as well as impairments in anticipatory postural adjustments (Viallet and others 1992), rhythm production (Halsband and others 1993), and bimanual coordination (Brinkman 1984). Also, characteristic symptoms of an SMA lesion are forced grasping and the alien hand sign, where one arm interferes with actions of the other (Goldberg and others 1981). This can be understood as an extreme example of bimanual coordination disorder. Likewise, human imaging studies have suggested substantial involvement of SMA in bimanual (Sadato and others 1997) and arm-leg (Debaere and others 2001) coordination.

Many of the aforementioned studies have supported the idea that SMA is responsible for the coordinated control of multiple joints and limbs. This view gains further support from its corticocortical and corticofugal connections. First, SMA receives dense projections from the primary (S1) and secondary (S2) somatosensory cortices, area 5, and the opercular part of area 7 in the posterior parietal cortex (Luppino and others 1993). Importantly, the hand area of SMA has a denser homotopic transcallosal connection when compared with M1 (Rouiller and others 1994). These connections allow SMA to access proprioceptive and haptic information from effectors on both sides, as well as information about their spatial relationship. This interhemispheric connection and bidirectional connection with ipsilateral M1 (Luppino and others 1993; Muakkassa and Strick 1979) allow each SMA to influence M1 in both hemispheres. Additionally, there is an overlap between the distribution areas of SMA neurons projecting to the forelimb and hindlimb representations in M1 (Hatanaka and others 2001). Regarding corticospinal projections, SMA neurons that project to distant segments of the spinal gray matter, including cervical and lumbar segments as well as upper and lower cervical segments, partially intermingle (He and others 1995). Last, SMA sends stronger projections to the pontomedullary reticular formation—a structure critical to postural control and interlimb coordination (Takakusaki and others 2016)—than M1 (Fregosi and others 2017).

By probing the area in action, single-neuron recordings provide clues to infer how SMA signals downstream structures to coordinate multiple joints and effectors. Tanji and others (1988) provided the first single-neuron evidence pointing to the role of SMA in bimanual coordination. They conducted recordings while monkeys pressed a key with the left, right, or both hands according to visual cues. While activity of most M1 neurons coincided with muscle activity of the contralateral hand, many SMA neurons exhibited patterns of discharge during the movement preparation and execution that could not be explained by muscle activity. Interestingly, a subset of SMA neurons was active only during the bimanual keypress, while another was active during movements of either hand in unimanual keypress but not in bimanual trials. The authors proposed that SMA is involved in the transformation of information about the instructed hand(s) into the pattern of neural activity for movement execution.

With regard to coordination of sequential movements, Shima and Tanji (2000) recorded neuronal activity in SMA while monkeys performed memorized unimanual motor sequences, which included three separate movements (push, pull, and turn a manipulandum). SMA neurons showed two types of characteristic activity: firing in between two particular movements (e.g., pull then turn) and firing before initiating a specific sequence. Critically, pharmacologic inactivation of bilateral SMA deteriorated performance in the task (Shima and Tanji 1998).

**Figure 3.**
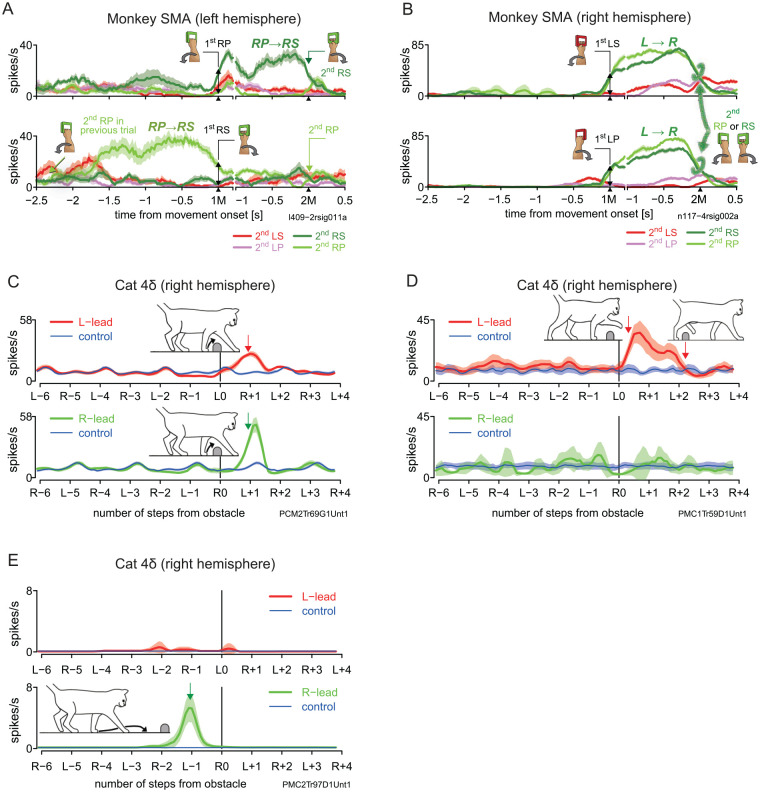
Single-neuron activity in the primate supplementary motor area (SMA) and the feline 4δ. (A) An SMA neuron shows preferentially increased activity during an interval beginning with right arm pronation (RP) and ending with supination of the same arm (RS). The basic display formats in panels A and B are the same as in Figure 2D. Note that in the bottom row the enhanced activity was preceded by the 2M (RP) in the previous trial. (B) An SMA neuron exhibits preferentially enhanced activity (traces indicated with green brackets) while awaiting right arm movement after the left arm movement regardless of action (pronation or supination). The activity following right arm movement was weaker (data not shown). Data are taken from Nakajima and others (2013). (C) A 4δ neuron moderately increases in activity in the period between the passage of the left and right forelimbs in the left-lead condition but strongly increases in the period between the passage of the right and left forelimbs in the right-lead condition. Modified from Figure 8F of Nakajima and others (2019). The basic display formats for panels C, D and E are the same as in the perievent histograms in Figure 2F. (D) A 4δ neuron exhibits selective activity enhancement in the period between the passage of the left forelimb and left hindlimb in the left-lead condition. Modified from Figure 2C of Nakajima, Fortier-Lebel, and Drew (2022). (E) A 4δ neuron that was active exclusively when the cat was about to place the left limb just in front of the obstacle. Modified from Figure 8C of Nakajima and others (2019). LP = left forearm pronation; LS = left forearm supination; RP = right forearm pronation; RS = right forearm supination.

Single-neuron recordings while monkeys performed multiple unimanual and bimanual motor sequences provide evidence that SMA supports switching of movement and/or effectors in motor sequences (Nakajima and others 2013). Indeed, characteristics of SMA were neuronal activity that appeared to link two different movements (Figure 3A) and activity related to the switching of arms (e.g., left arm then right) regardless of action (pronation or supination; Figure 3B). The combined evidence of single-neuron activity and connectivity of SMA with other sensorimotor areas (areas 5, S1, S2, and M1) suggests that SMA prepares each task-required movement under somatosensory guidance and in reference to the preceding movement, thus permitting the control of bimanual coordination and planned motor sequences.

Evidently, interlimb coordination is critical to voluntary behaviors beyond bimanual tasks. While walking on uneven ground, animals must precisely control each step and coordinate successive steps made with different limbs. In humans, while many case reports indicate that SMA is essential for normal walking (e.g., Della Sala 2002), it also seems to play a role in coordinating voluntary steps. SMA activity increases when a foot must be placed at a precise position (Koenraadt and others 2014) or when the subjects mentally imagine negotiating an obstacle while walking (Wang and others 2009). Nordin and others (2019) recently identified transient enhancement of oscillatory activity in SMA before subjects stepped over an obstacle.

Given scant reports on single-neuron activity in the primate SMA while walking, introducing knowledge obtained about area 4δ in the cat, a premotor area spanning both banks of the cruciate sulcus (Figure 1B), is of interest. This area shares several similarities with primate SMA. Anatomically, 4δ is bidirectionally connected with 4γ (Ghosh 1997a). Nakajima, Fortier-Lebel, and Drew (2022) also reported intermingling of the parts of 4δ that project to the forelimb and hindlimb representations of 4γ, suggesting a potential role in forelimb-hindlimb coordination alongside 4δ’s own efferent to the spinal cord (Ghosh 1997b) and to the pontomedullary reticular formation (Rho and others 1997). Furthermore, 4δ, like SMA, receives dense projections from area 5 and hindlimb representation in S1. Regarding microstimulation, 4δ requires a stronger current to induce movement than area 4γ but often evokes a substantial effect in the forelimb and hindlimb, drawing further parallels with the primate SMA (Fortier-Lebel and others 2021).

Our recent single-neuron recordings have shown evidence that 4δ is responsible for interlimb coordination (Nakajima, Fortier-Lebel, and Drew 2022; Nakajima and others 2019). Figure 3C shows a neuron that fired between two voluntary steps with both forelimbs, with greater activity when the left forelimb led (top row). Another sample neuron shown in Figure 3D fired continuously from the moment that the left forelimb began to step over the obstacle until the left hindlimb did so. The activities in Figure 3A and 3B and Figure 3C and 3D were recorded from separate animal species performing different types of motor actions, yet they share the similarity of occurring between two elements of a specific movement sequence.

The view that area 4δ is responsible for interlimb coordination is strengthened by activity occurring even before the steps over the obstacle. The neuron shown in Figure 3E fired exclusively when the cat was about to place its left forepaw in front of the obstacle. The plant paw must be placed at an appropriate distance from the obstacle for smooth clearance (Lajoie and Drew 2007). Such accurate paw placement depends on a voluntary adjustment of stride length, which entails controlling both the propulsive forces generated by the stance limbs and the pendular movement of the swing limb. Thus, area 4δ appears to orchestrate interlimb coordination starting from at least one step before the obstacle down to the final clearance by the hindlimbs. With similarities in anatomic and functional connectivity, primate SMA and feline 4δ may be analogous structures capable of coordinating multiple effectors, as suggested by Nakajima and others (2019; Nakajima, Fortier-Lebel, and Drew 2022). Presumably, the precursor of SMA in primate ancestors evolved the capacity to orchestrate complex coordination of limbs and joints, enabling movements in trees and eventually tool use.

## Cortical Regions Implicated in Action Inhibition: Primate Pre-SMA and Feline 6iffu

Pre-SMA sits rostrally adjacent to SMA (Figure 1A). Many studies in humans have implicated pre-SMA in motor inhibition, sometimes with SMA (Filevich and others 2012). For example, electrical stimulation in pre-SMA arrests ongoing body movements (Lüders and others 1995). When response conflict exists, pre-SMA is tonically active (Garavan and others 2003), and a patient with a focal lesion in pre-SMA was unable to inhibit competing single motor plans (Nachev and others 2007).

Microstimulation studies on primates have rather emphasized difficulty in evoking movement; pre-SMA requires a longer train or higher amplitude than SMA to induce movements. The effect is often slow onset and complex, involving multiple joints (Luppino and others 1991; Matsuzaka and others 1992). Interestingly, microstimulation coinciding with natural arm movements typically terminated the ongoing movement, redirecting the arm to a new position (Luppino and others 1991; cf., Lüders and others 1995).

Difficulty in inducing movements from pre-SMA aligns with its connectivity: unlike SMA, pre-SMA does not connect directly with M1 (Luppino and others 1993) and has few direct projections to the spinal cord (Dum and Strick 1991). The paucity of response to somatosensory stimuli also distinguishes pre-SMA from SMA. In contrast, pre-SMA receives projections from parietal area 7 and the dorsolateral prefrontal cortex (Luppino and others 1993), allowing the area to access visual and contextual information.

In terms of single-neuron activity, the contrast between pre-SMA and SMA is evident in simple delayed reaching tasks (Matsuzaka and others 1992) and reaching-grasping tasks (Rizzolatti and others 1990). Specifically, phasic activity synchronized with action execution is less frequent than in SMA. Rather, a more prevalent feature of pre-SMA neurons is sustained, anticipatory activity, often beginning during the delay period and continuing until the action is allowed (Matsuzaka and others 1992). During action execution, neuronal activity is often suppressed (Hoshi and Tanji 2004; Rizzolatti and others 1990), implying that pre-SMA disinhibits the action in a timely manner. This view has been supported by observations of Mita and others (2009). To investigate the mechanism of internal timing of action execution by pre-SMA, the authors devised a task in which monkeys had to hold a key for a specified interval (2, 4, or 8 seconds) before releasing it to receive a reward. They found that a group of neurons in pre-SMA continued to increase their firing rate throughout the interval, which then rapidly decreased before action onset. Such activity could serve to withhold an action until the appropriate time.

Inhibition is essential for switching between two actions as it involves inhibiting the previous motor plan and facilitating the next. To investigate the role of pre-SMA in action switching, Matsuzaka and Tanji (1996) and Isoda and Hikosaka (2007) trained monkeys to perform a button-pressing task and an oculomotor task, respectively. In both tasks, an action-reward contingency was maintained for several trials. Then, an auditory or visual cue informed the monkeys of contingency change. The authors found that many pre-SMA neurons increased their activity when the monkey was going to successfully switch to the alternative action in response to the cue change. Absence of the activity increase correlated with an erroneous action choice in favor of the previous contingency. Interestingly, the error was followed by a belated activity increase. Furthermore, Isoda and Hikosaka (2007) found that the activities of switch-related neurons were heterogeneous—some were thought to inhibit an action, others to facilitate the alternative, and the remaining to engage in inhibition and facilitation. Isoda and Hikosaka (2008) extended the recording to the subthalamic nucleus and suggested that signals from pre-SMA act to suppress habitual actions through a hyperdirect pathway (Nambu and others 2002).

Other studies detected pre-SMA activity triggered by errors in one’s own actions (Fu and others 2019) or even others’ actions (Yoshida and others 2012). Such error-related activity could help shape future actions. It is therefore plausible for pre-SMA activity to increase during learning through trial and error and decrease as learning progresses (Nakamura and others 1998; see Nachev and others 2008).

From these observations, a reasonable interpretation may be that seemingly distinct motor-cognitive functions—inhibition, facilitation, switching, performance monitoring, and learning—are in part similar processes that commonly involve pre-SMA. Furthermore, such processes are necessary for performing a sequence of actions in the correct order. For example, upon starting a car, hastily driving should be withheld before fastening the seatbelt. In longer sequences guided by memory, monitoring progress becomes critical. Coherently, bilateral inactivation of pre-SMA renders monkeys unable to perform motor sequences from memory, as was the case with SMA (Shima and Tanji 1998). Later, Shima and Tanji (2000) showed that pre-SMA neurons, more frequently than SMA neurons, represent the ordinal position of actions in a sequence performed by the monkey, irrespective of the action’s specific details (see Clower and Alexander 1998). This implies that pre-SMA is more involved in monitoring a sequence’s progress than SMA.

**Figure 4.**
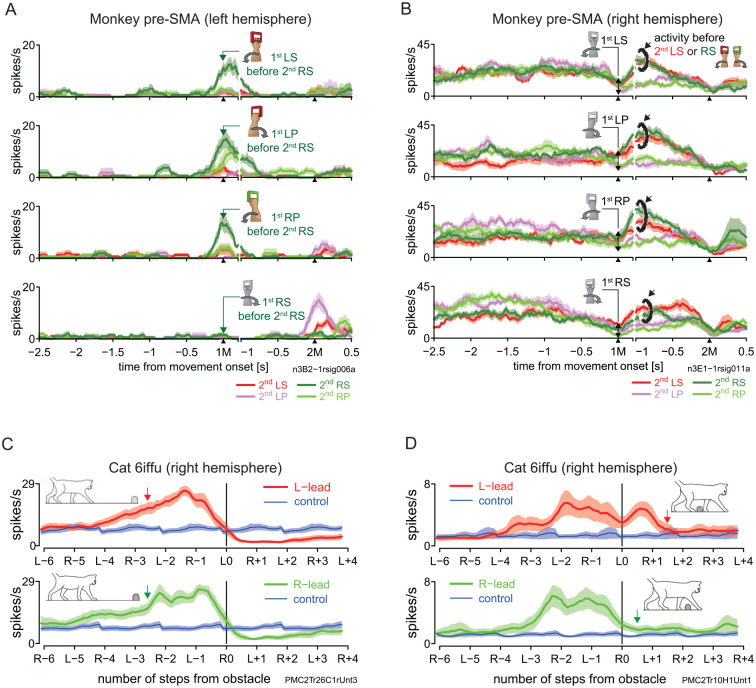
Single-neuron activity in the primate pre-SMA and the feline 6iffu. (A) A sample pre-SMA neuron exhibits phasic activity during the performance of any movement other than right forearm supination (RS) in the first movement (1M) when the second movement (2M) was RS. Note a virtually complete lack of activity when RS was to be repeated (bottom row). The basic display formats for panels A and B are the same as in Figure 2D. Modified from Figure 8A of Nakajima, Hosaka, and Mushiake (2022). (B) A pre-SMA neuron exhibits activity enhancement (traces with black brackets) after the 1M when the 2M was supination of either arm. Modified from Figure 7C of Nakajima, Hosaka, and Mushiake (2022). (C) A neuron in 6iffu exhibits an increase in activity that started more than four steps before the obstacle regardless of which forelimb was the first to step over the obstacle. The activity terminated when the lead forelimb left the treadmill surface to step over the obstacle in either condition. The basic display formats for panels C and D are the same as in the perievent histograms in Figure 2F. Modified from Figure 14A of Nakajima and others (2019). (D) Another example of a neuron with an early activity onset before the obstacle, which continued until the right forelimb stepped over the obstacle in either condition. Modified from Figure 14C of Nakajima and others (2019). LP = left forearm pronation; LS = left forearm supination; RP = right forearm pronation; RS = right forearm supination; SMA = supplementary motor area.

Nakajima, Hosaka, and Mushiake (2022) recently provided evidence that pre-SMA orchestrates motor sequences through inhibition and switching, using a task almost identical to Nakajima and others (2013; see the previous section). They identified neurons showing phasic activity, indicating a preference for specific second movements (2Ms) while performing memorized motor sequences. Figure 4A shows a sample neuron preferring right forearm supination for the 2M. Notably, timing of the activity was tied not to the 2M but to the first movement (1M; Figure 4A, top three rows), suggesting a timely inhibition to prevent premature execution of the 2M. In agreement, this activity was attenuated in cases of premature execution. Moreover, such activity was absent in repetition sequences, where the inhibition was unnecessary (Figure 4A, bottom). Another neuron exhibited phasic activity following the 1M when the 2M was supination of either arm, consistent with pre-SMA signaling the switch to a specific action independent of effector (Figure 4B). Such effector-independent activity in pre-SMA has been reported elsewhere (Hoshi and Tanji 2004; Nakajima and others 2013). In summary, pre-SMA’s cardinal roles include inhibiting actions associated with a goal or reward due to repetition or temporal proximity, switching to alternative actions as needed, and monitoring actions.

One would expect that voluntary gait modifications are subject to inhibition-based control. As there is currently no literature on pre-SMA neuronal activity in walking humans or primates, evidence from its analogous brain region in cats provides clues to speculate about the roles of pre-SMA in controlling locomotion. We have suggested that area 6iffu in the cat, located deep in the ventral bank of the cruciate sulcus (Figure 1B), is analogous to the primate pre-SMA. Similarities to pre-SMA are the sparse projections to the spinal cord or area 4γ (M1), the difficulty in inducing movement with microstimulation (Fortier-Lebel and others 2021), and the sensitivity to visual stimuli, presumably via its connections with parietal area 7 (Nakajima and others 2019).

When area 6iffu in walking cats was recorded (Nakajima and others 2019), many neurons increased their activity several steps before the obstacle yet without correlation with ongoing cyclic muscle activity. In most of these neurons, the activity pattern did not differ between left- and right-lead conditions (defined in Figure 2F and G), as shown in Figure 4C. The activity then dropped below the baseline when either forelimb began to step over the obstacle. Activity elevation in 6iffu neurons thus appears to match the closing spatiotemporal relationship between the obstacle and the cat.

Paralleling pre-SMA in reaching primates, we suggest that 6iffu exerts an inhibitory control to determine when to modify a step to negotiate the obstacle, although it has yet to be determined whether feline 6iffu has access to a hyperdirect pathway. The activity elevation in Figure 4C could withhold the initiation of the modified step while its subsequent attenuation could disinhibit that step. We furthermore suggest that 6iffu is involved in computations to determine which forelimb will step over the obstacle first. In 6iffu, we found a group of neurons that changed their activity duration depending on which forelimb was leading. A sample neuron in Figure 4D ceased firing when the right forelimb began to step over the obstacle first (bottom row) but remained active when the left forelimb led the other (top). Thus, feline 6iffu appears to be involved in determining when and with which forelimb a modified step should be taken based on spatiotemporal information.

## Visuomotor Association and Goal-Oriented Motor Representation: Primate PMd

PMd is a lateral division of the primate area 6, bordered medially by SMA and caudally by M1 (Figure 1A; Geyer and others 2000). PMd contains a lower limb, trunk, and proximal and distal upper limb motor representation along its caudomedial-to-rostrolateral extent. It requires comparable or slightly higher current amplitudes than SMA to elicit movements but displays a simpler motor representation, with a much lower proportion of sites evoking complex noncontiguous joint movements than SMA (Fogassi and others 1999; Raos and others 2003).

Like SMA, PMd possesses its own access to descending motor pathways, projecting strongly to the cervical and lumbar spinal cord (He and others 1993; Morecraft and others 2019), as well as to the pontomedullary reticular formation (Fregosi and others 2017; Keizer and Kuypers 1989). It is also densely interconnected with M1 (Dum and Strick 2005; Hatanaka and others 2001).

PMd is characterized by strong visual and somatosensory inputs originating from area 5 of the posterior parietal cortex and is notably the main target of the medial intraparietal area, which lies in the sulcus that separates areas 5 and 7 and is itself involved in the visuospatial processing of targets during reaching (Matelli and others 1998; Pesaran and others 2006; Wise and others 1997). Finally, PMd communicates bidirectionally with SMA (Luppino and others 1993) and, mainly through its rostral portion, with pre-SMA and the prefrontal cortex (Luppino and others 1993; Luppino and others 2003).

Specific interest in PMd was simultaneously prompted by (1) the visuomotor association deficits that could be observed following its bilateral ablation in the primate (though at the time subsumed in the “lateral premotor cortex”; Halsband and Passingham 1982) and (2) the relationship of its single-neuron activity with visuospatial features of reaching tasks (Weinrich and Wise 1982). Subsequent studies on PMd activity have mainly investigated its contribution to single arm movements. These have converged on a role in action selection based on arbitrary cues (Cisek and Kalaska 2005; Yamagata and others 2009; see Wise and others 1997) and in guidance of the effector in reference to visuospatial information (Pesaran and others 2006; Raos and others 2004).

**Figure 5.**
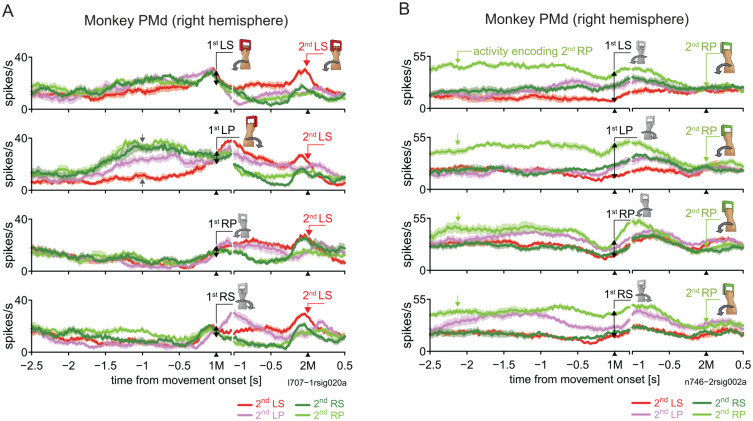
Single-neuron activity in the primate dorsal premotor area (PMd). (A) A typical example of a PMd neuron exhibiting preparatory activity prior to movement with the left arm. Paired gray arrows indicate the range of varying activity levels with respect to the second movement (2M) that occurred before execution of the first movement (1M; LP). Modified from Figure 4C in Nakajima, Hosaka, and Mushiake (2022). The basic display formats for panels A and B are the same as in Figure 2D. (B) A PMd neuron shows tonic activity selective for a particular 2M (RP) before execution of the 1M (lime trace). Modified from Figure 3 of Nakajima, Hosaka, and Mushiake (2022). LP = left forearm pronation; LS = left forearm supination; RP = right forearm pronation; RS = right forearm supination.

While some authors have proposed a specific contribution of PMd to internally generated movement sequences (Ohbayashi and others 2016), we emphasize that the functions outlined here remain important to sequences in general. Indeed, these may be guided step-by-step from extraneous cues or require selection of the final desired action from such cues before contextually working out the sequence leading to its realization. Comparing the activity of PMd neurons with that of pre-SMA in the same sequential bimanual experiment discussed in the previous section, Nakajima, Hosaka, and Mushiake (2022) showed that in visually guided trials, for most PMd neurons, instructional cues triggered activity selective for a specific forthcoming movement, irrespective of its rank in the sequence. During memory-guided trials, this selective activity emerged spontaneously. Figure 5A shows a sample neuron displaying a left arm preference for the forthcoming movement. Such step-by-step rank-indiscriminate neurons formed the largest group in PMd, were significantly more prevalent than in pre-SMA, and may participate in controlling each movement. Notably, the activity prior to left arm pronation as the 1M was influenced by the 2M. This suggests that PMd exerts varying levels of facilitation or suppression on downstream motor structures for the appropriate performance of the 1M as a function of the 2M.

Interestingly, the second-largest group of PMd neurons showed another defining sequence-relevant feature: in memory-guided trials, once the sequence was learned, PMd neurons preferentially represented the 2M in the instructed sequence significantly more often than pre-SMA. Strikingly, nearly half of that neuron population already exhibited activity selective for the 2M even before onset of the 1M. A representative neuron is shown in Figure 5B. The selective activity of such neurons often terminated a few hundred milliseconds before the 2M. Because visuospatial factors were minimized in memory-guided trials, we suggest that this activity signals the movement most associated with the reward (i.e., the end goal of the sequence). Furthermore, because the discharges began before sequence execution, in a natural setting with a longer sequence of actions, their early signaling of the final action would inform which lead-up sequence to orchestrate. Early termination of the activity supports this idea, as well as the possibility that the activity inhibited the movement linked to the reward until the appropriate time.

As with SMA and pre-SMA, little to no information on primate PMd is available at the single-neuron level during locomotion. Beyond a report of rhythmic activity during simple quadrupedal treadmill walking (Foster and others 2014), the most relevant study is that of Berger and others (2020). In their task, a monkey had to freely walk and reach to one of several targets following a visual cue. They showed that neurons in PMd already encoded the intended reach at a distance. As in the work of Nakajima, Hosaka, and Mushiake (2022), this would suggest that PMd signals the end goal early on, allowing determination of the sequence of movements that realize it. In the sole context of locomotion, intending to step over an approaching obstacle in a particular way also requires the orchestration of a lead-up gait sequence.

In cats, anatomic and microstimulation evidence suggests analogies between area 6a—containing areas 6aα, 6aβ, and 6aγ in Figure 1B—and the primate lateral premotor areas (Fortier-Lebel and others 2021; Ghosh 1997b), with 6aα in particular being most coherent with PMd. Applied during locomotion, microstimulation of area 6aα shows often-strong responses in the forelimb and hindlimb, well integrated to the phase of the step cycle, indicating a significant capability to modify gait (Fortier-Lebel and others 2021). At this time, the only report of 6a activity during locomotion comes from Viana Di Prisco and others (2023), pooling 6aα and 6aγ neurons. Comparing overground locomotion with stepping on horizontal ladders and over a series of barriers, they showed that most single neurons displayed rhythmic activity during overground walking and that, in most of them, this activity was modulated by the two visually guided conditions in the timing of peak activity, the depth of modulation, or both. Whether such changes contribute to the selection and/or guidance of the most appropriate steps remains to be elucidated.

## Concluding Remarks

**Figure 6.**
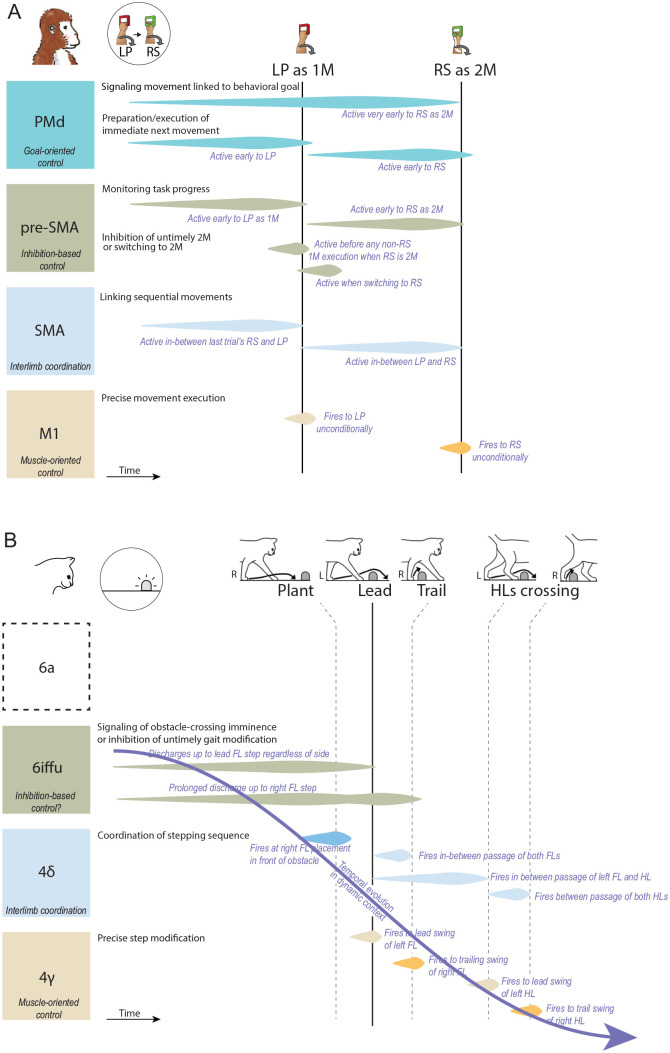
Summary depiction of single-neuron activity in multiple motor areas during the performance of a bimanual sequence task (monkey) and gait modification task (cat). (A) Motor areas of the primate involved in voluntary movement sequences. In this example, an arbitrary sequence of “LP-RS” is used to depict characteristic activity conditionalities. The vertical lines represent the two movement onsets of the sequence, with each row of activity (depicted, for simplicity, as a generic ramping shape) showcasing a different neuron. Interpretation of the neuron’s (or neurons’) potential contribution to the task is provided on the upper left. Objective conditionality of the activity is reported in pale italics. (B) Motor areas of the cat involved in voluntary gait modifications. In this example, the animal steps over an obstacle, leading with the left limb. As in panel A, each row represents a different neuron. Vertical lines depict the onset of important steps along the obstacle negotiation sequence. Obstructed locomotion is a dynamic task in which the features and relative location of the obstacle are integrated into the motor plan as a function of the approach; a curved arrow depicts the evolution at a behavioral timescale of the obstacle negotiation plan. Information on area 6a with regard to locomotor planning is currently lacking. In panels A and B, all neurons are located in the right hemisphere, except for the darker shaded neurons in 4δ and 4γ/M1, whose activity is consistent with the left hemisphere. Note that while each cortical area contains varied populations of cells, including some displaying simple movement-related discharges, here we schematize single-neuron activity characteristic of each cortical area that is discriminative and relevant to the conditions of the task. 1M = first movement; 2M = second movement; FL = forelimb; HL = hindlimb; L = left limb; LP = left forearm pronation; LS = left forearm supination; PMd = dorsal premotor area; R = right limb; RP = right forearm pronation; RS = right forearm supination; SMA = supplementary motor area. Other abbreviations as in Figure 2.

Our understanding of how multiple motor areas contribute to controlling voluntary movement has been shaped by decades of intense human and primate research focused on the planning and orchestrated execution of our rich upper-limb movement repertoire. Evidence from single-neuron recordings in walking cats strongly suggests that voluntary gait modification also relies on the involvement of multiple motor areas. This unique evidence from another flexible motor behavior, locomotion, with that of cross-species functional analogies prompts us to suggest that motor areas contribute in fundamentally similar ways to different motor behaviors, providing the same building blocks that form complex goal-oriented motion in gait and arm movement (Figure 6). It prompts us to reassess the lack of current primate data over gait control. Indeed, while high-resolution neural activity studies in the primate have very recently concerned gait beyond steady locomotion (Berger and others 2020; Xing and others 2022), further recordings across the frontoparietal cortex of the primate during gait modification are necessary to verify this proposition and determine the extent of our suggested cross-species areal analogies. Translationally, given the clear changes in neuronal activity that we observed in the cat, investigating whether upcoming gait modifications can be decoded from SMA, pre-SMA, and other premotor areas (or their cat equivalents) could be highly relevant for the development of locomotor neuroprosthetics.
